# ceRNA Cross-Talk in Paulownia Witches’ Broom Disease

**DOI:** 10.3390/ijms19082463

**Published:** 2018-08-20

**Authors:** Guoqiang Fan, Zhe Wang, Xiaoqiao Zhai, Yabing Cao

**Affiliations:** 1 Institute of Paulownia, Henan Agricultural University, Zhengzhou 450002, China; wangzhe6636@foxmail.com (Z.W.); cyb201406@163.com (Y.C.); 2 College of Forestry, Henan Agricultural University, Zhengzhou 450002, China; 3 Henan Academy of Forestry, Zhengzhou 450008, China; user7117@163.com

**Keywords:** lncRNA, circRNA, miRNA, mRNA, PaWB

## Abstract

Long noncoding RNA (lncRNA), circular RNA (circRNA), and microRNA (miRNA) are important in the regulation of life activities. However, their function is unclear in *Paulownia fortunei*. To identify lncRNAs, circRNAs, and miRNA, and investigate their roles in the infection progress of Paulownia witches’ broom (PaWB) disease, we performed RNA sequencing of healthy and infected *P. fortunei*. A total of 3126 lncRNAs, 1634 circRNAs, and 550 miRNAs were identified. Among them, 229 lncRNAs, 65 circRNAs, and 65 miRNAs were differentially expressed in a significant manner. We constructed a competing endogenous RNA (ceRNA) network, which contains 5 miRNAs, 4 circRNAs, 5 lncRNAs, and 15 mRNAs, all of which were differentially expressed between healthy and infected *P. fortunei.* This study provides the first catalog of candidate ceRNAs in Paulownia and gives a revealing insight into the molecular mechanism responsible for PaWB.

## 1. Introduction

Paulownia species are cultivated in several temperate zones worldwide for their rapid growth and ability to adapt to extreme environments [1,2]. Paulownia wood has been used for making furniture, making violins, as building material, and for land reclamation [3]. However, Paulownia witches’ broom (PaWB) disease has seriously threatened the production of Paulownia. PaWB is caused by phytoplasmas that belong to the Aster Yellows group *Candidatus* Phytoplasma asteris (16SrI-D) and are spread by insect vectors [4]. PaWB has been the focus of many studies, and some PaWB-related genes [5,6,7,8,9,10], microRNAs (miRNAs) [11,12,13,14], proteins [15,16,17], metabolites [18], and long noncoding RNAs (lncRNAs) [19] have been revealed. However, the underlying mechanisms of PaWB are still not known.

Lately, a competing endogenous RNA (ceRNA) hypothesis has been put forward which exhibits an interesting relationship among mRNA, lncRNA, circRNA, and pseudogene transcripts. In other words, these RNA transcripts would bind to miRNA to influence the gene expression [20]. Accumulating data have demonstrated that ceRNAs are present in plants and could be implicated in plant responses to biotic and abiotic stresses. In maize, long intergenic noncoding RNAs were shown to participate in a layer of regulatory interactions as miRNA targets or decoys [21]. A ceRNA network was constructed in rice and may be involved in the biological response to inorganic phosphate starvation [22]. In Populus, a ceRNA network has been found, and its function in the adaptation to low nutrition was revealed [23].

Here, we identified expression changes of mRNAs, lncRNAs, circRNAs, and miRNAs between healthy and infected *Paulownia fortunei*, and constructed a ceRNA network to illuminate the underlying mechanisms of PaWB. Our findings reveal a novel mechanism for PaWB.

## 2. Results

### 2.1. Morphology of Heathy and Infected Paulownia Fortunei Seedlings

**Figure 1:**
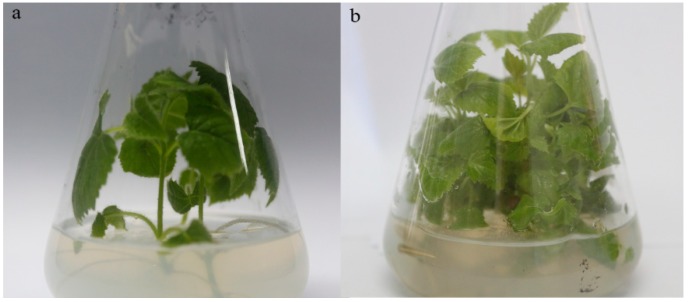
Morphology of healthy and infected plantlets. (**a**) heathy *P. fortunei*, (**b**) infected *P. fortunei*.

Unlike the heathy *P. fortunei* (PF), the infected *P. fortunei* (PFI) showed disease symptoms, most prominently witches’ broom (Figure 1). Nested PCRs detected phytoplasma sequences in PFI, and not in PF (Figure S1). This result is consistent with previous studies [5,6,15].

### 2.2. RNA Sequencing and Identification of miRNAs, mRNAs, lncRNAs, and circRNAs

To obtain a comprehensive view of the miRNA repertoire in Paulownia, we constructed and sequenced six sRNA libraries (PF-1,2,3 and PFI-1,2,3). A total of 46 million reads were obtained (Table S2). The sRNA data were processed and mapped to the Paulownia genome (http://paulownia.genomics.cn), and miRNAs were annotated according to a well-established protocol. A total of 550 miRNAs (395 conserved and 155 novel) were identified (Table S3). We identified members of the 60 miRNA families that are conserved in plants and also identified novel Paulownia-specific miRNAs. A total of 126 miRNAs families were characterized as novel miRNAs and were named Pf-mir1 to Pf-mir126 (Table S4). One-third of the novel miRNAs (47 miRNAs) were 21 nt in length.

We performed whole transcriptome sequencing of the six RNA libraries constructed from PF and PFI with biological replicates (*n* = 3 biological replicates per material). A total of 486 million paired-end reads were obtained (Table S2). After stringent filtering, the clean reads were aligned to the Paulownia reference genome. The mapping rate for PF (71.3%) sequences was higher than for PFI (59.3%) sequences (Table S2), probably because the reference genome was derived from healthy *P. fortunei*. The high-quality clean reads were subjected to an optimized pipeline to identify circRNAs, lncRNAs, and mRNAs. A total of 32,086 mRNAs (30,427 (PF) and 30,856 (PFI)) were identified (Table S3). Approximately 3126 lncRNAs were obtained from the PF and PFI libraries, and an additional 382 and 314 lncRNAs were found expressed only in the PF and PFI libraries, respectively (Table S3). According to the locations of lncRNAs in the reference genome, we identified 1716 and 1632 intergenic transcripts; 210 and 248 intronic transcripts; 754 and 730 exonic overlaps with a reference transcript on the opposite strand; 17 and 17 generic exonic overlaps with a reference transcript; and 115 and 117 potentially novel isoforms in the PF and PFI libraries, respectively. We filtered out the junction reads with non-canonical splice sites or cross genes alignments and obtained about 8 million back-spliced junction reads for identification of candidate circRNAs (Table S2). Finally, we identified a total of 1634 circRNAs (Table S3).

### 2.3. Expression Analysis of miRNAs, circRNAs, lncRNAs, and mRNAs

**Figure 2:**
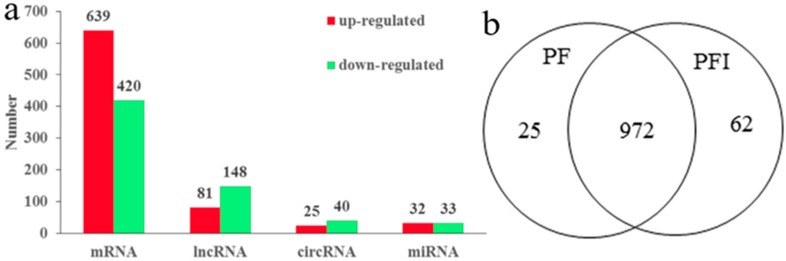
Expression analysis of microRNAs (miRNAs), Circular RNAs (circRNAs), long noncoding RNAs (lncRNAs), and mRNAs. (**a**) Differently expressed miRNA, mRNA, lncRNAs and circRNAs; (**b**) common and specific differentially expressed genes (DEGs) in PF and PFI.

A total of 65 (48 conserved and 17 novel) differentially expressed miRNAs (DEMs) were identified (*p* value < 0.05 and fold change >2 or <−2), including 32 (25 conserved and 7 novel) up-regulated and 33 (23 conserved and 10 novel) down-regulated miRNAs (Table S4 and Figure 2a). We predicted 850 target genes for the 65 DEMs. 

A total of 1059 genes were differentially expressed in a significant way (639 up-regulated and 420 down-regulated) in the PF samples compared with the PFI samples, and were considered as candidate PaWB-related genes (Table S4 and Figure 2a). A Venn diagram of the differentially expressed genes (DEGs) shows the 25 PF-specific, 62 PFI-specific, and 972 common genes that were differentially expressed between the PF and PFI samples (Figure 2b). The functional analysis mapped the DEGs to 129 Kyoto Encyclopedia of Genes and Genomes (KEGG) pathways. Among them, “Phenylpropanoid biosynthesis”, “Carbon fixation in photosynthetic organisms”, “Tropane, piperidine, and pyridine alkaloid biosynthesis”, “Metabolism of xenobiotics by cytochrome P450”, “Isoquinoline alkaloid biosynthesis”, “Tyrosine metabolism”, “Selenocompound metabolism”, and “Monoterpenoid biosynthesis” were significantly enriched (Table S5). In the GO analyses, 25 biological process, 15 cellular components, and 10 molecular function terms were significantly enriched (Figure S2).

A total of 229 differentially expressed lncRNAs (DELs) were identified: 81 were up-regulated and 148 were down-regulated (Table S4 and Figure 2a). We found more DELs (7.3%) than DEGs (3.3%) between the PF and PFI libraries, suggesting that the lncRNAs and protein-coding genes may have markedly differential expression patterns in the PF and PFI samples. We also found 14 and 27 DELs that were expressed specifically in PF and PFI samples, respectively (Table S4). Previous studies showed that lncRNAs are preferentially located in close proximity to the genes that they regulate. A total of 1048 target genes were predicted for the 229 DELs (Table S4). 

The expression levels of the circRNAs were determined by the number of identified reads adding one normalized by the total number of reads in each library. A total of 65 differentially expressed circRNAs (DECs) were detected between the PF and PFI libraries (Table S4 and Figure 2a). The principal component analysis of differentially regulated miRNA, mRNA, lncRNAs, and circRNAs in PI and PFI was shown in Figure S3.

### 2.4. Properties of mRNAs, lncRNAs and circRNAs

We compared the number, expression level, and length distribution of the identified mRNAs, lncRNAs, and circRNAs. The number and expression levels of the mRNAs were higher than those of the lncRNAs and circRNAs (Figure S4). The length distributions of the lncRNAs and circRNAs were similar, and the mRNAs were longer (Figure S4). 

### 2.5. Candidate PaWB-Related miRNAs, mRNAs, lncRNAs, and circRNAs

Among the 850 target genes for the 65 DEMs, about 38 genes, which were targeted by 15 DEMs, were differentially expressed. We considered these 15 DEMs as candidate PaWB-related miRNAs. In mulberry, miR156/160/172/395 were identified as phytoplasma-responsive miRNAs [24]. In Mexican lime, miR156/160/169 changed in response to phytoplasma infection [25]. In jujuba, miR156/159/160/172/395 were involved in the response to phytoplasma infection [26]. In our previous study, miR169 were among the common PaWB-related miRNAs in *P. fortunei*, *P. tomentosa*, and *P. tomentosa* × *P. fortunei* [27]. The target genes of miR169, Nuclear factor YA1 (NFYA1), NFYA7, and NFYA10, were also found in *Populus tomentosa* under fungus stress [28].

In this study, 1059 DEGs were considered as candidate PaWB-related genes. The KEGG analysis for the 1059 DEGs showed that the pathways “Circadian rhythm–plant”, “Diterpenoid biosynthesis”, “Endocytosis”, “Flavonoid biosynthesis”, “Limonene and pinene degradation”, “Phenylpropanoid biosynthesis”, “Stilbenoid, diarylheptanoid and gingerol biosynthesis”, “Plant–pathogen interaction”, “Pyrimidine metabolism”, “Ribosome”, and “Plant hormone signal transduction” were common in our previous study [27]. In lime, 43 metabolic and regulatory pathways were highly enriched for the DEGs in healthy and infected libraries [29]. Among these 43 pathways, 37 pathways were found in the present study.

Among the 1048 target genes of the 229 DELs, 1039 genes, which were targeted by 229 DELs, were differentially expressed. To identified candidate PaWB-related lncRNAs, we looked for lncRNAs target genes that were candidate PaWB-related genes. Finally, we identified 229 candidate PaWB-related lncRNAs.

Among the 62 circRNA host genes of the 65 DECs, 9 host genes of 10 DECs were differentially expressed. These 10 DECs were considered as candidate PaWB-related circRNAs. Auxin-induced protein PCNT115-like isoform (*AIP*, EVM0004162.1) was host gene of circRNA200 and circRNA362. *AIP* is related to plant defense and auxin-mediated plant growth [30] and *AIP* was down-regulated in response to phytoplasma infection [31]. 12-oxophytodienoic acid reductase (*OPR*, EVM0008613.1) was the host gene of circRNA137. *OPR* encodes the enzyme involved in the last committed step in the octadecanoid pathway leading to jasmonic acid biosynthesis [32]. *OPR* plays a regulatory role in rice plant defense/stress response pathways [33]. Probable protein phosphatase 2C 40 (*PP2C*, EVM0027445.1) was the host gene of circRNA962 and is a key player in plant signal transduction processes [34]. Cinnamyl-alcohol dehydrogenase (*CAD*, EVM0012048.1) was the host gene of circRNA104 and plays an important role in the lignin biosynthetic pathway [35]. Long-chain acyl-CoA synthetase (*ACSL*, EVM0013816.1) was the host gene of circRNA1225 and is involved in fatty acid metabolism, which has been found to be influenced by phytoplasma infection [9]. Secologanin synthase-like (*Ses*, EVM0016611.1) was the host gene of circRNA770. *Ses* belongs to the oxidoreductase family and participates in indole and ipecac alkaloid biosynthesis [36]. Glutathione peroxidase (*gpx*, EVM0019662.1) was the host gene of circRNA337; it is induced by oxidative stress and plays specific roles in scavenging reactive oxygen species [37]. Chlorophyllide a oxygenase (*CAO*, EVM0016969.1) was the host gene of circRNA345 and is involved in porphyrin and chlorophyll metabolism [38]. 

### 2.6. The ceRNA Network

**Figure 3:**
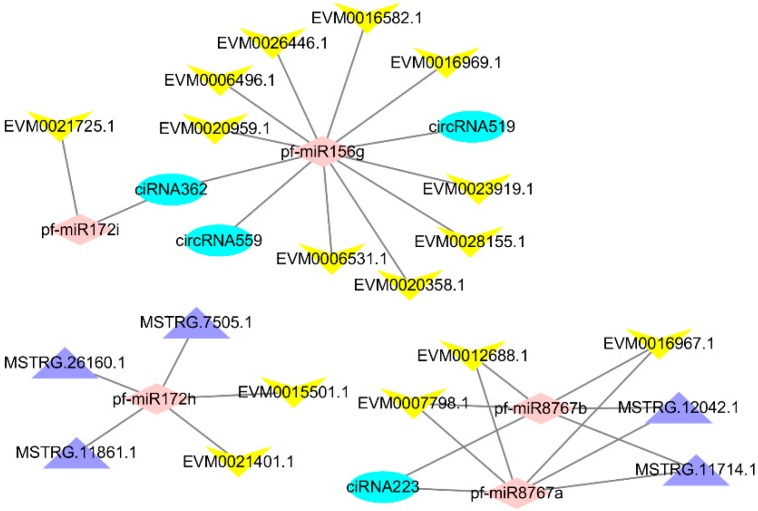
The Constructed ceRNA network. pink: miRNA, purple: lncRNA, blue: circRNA, yellow: mRNA.

Several studies have indicated that lncRNAs and circRNAs could also be targeted by miRNAs in plants. A large number of miRNAs have been identified in this and previous studies. To systematically investigate the miRNA-mediated regulatory mechanism of lncRNAs and circRNA in Paulownia, target mimics were applied to predict miRNA targets among 1059 DEGs, 229 DELs, and 65 DECs. The ceRNA networks of lncRNA–miRNA–mRNA and circRNA–miRNA–mRNA were integrated from the relationships between lncRNA–miRNA, circRNA–miRNA, and mRNA–miRNA using Perl scripts. The ceRNA network was visualized by importing the above interactions into the Cytoscape software to assemble the regulatory ceRNA network (Figure 3). The ceRNA network has three parts, with each part centered on pf-miR156j and pf-miR172i, pf-miR8767a/b, or pf-miR172h/k.

### 2.7. Verification of Seq-Results

**Figure 4:**
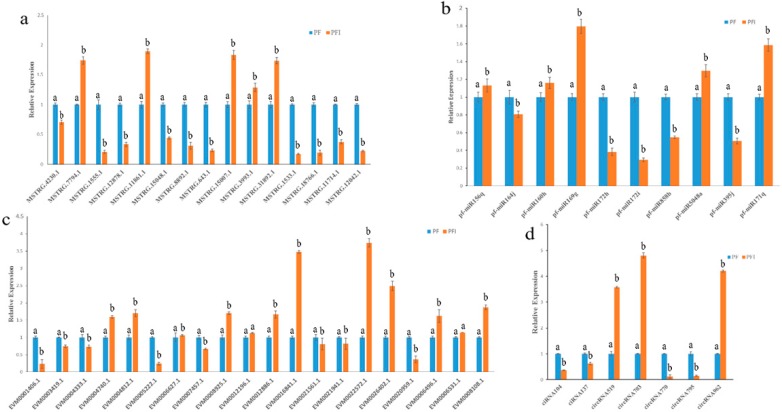
Quantitative Real-Time PCR (qRT-PCR) analysis. (**a**) Relative expression levels of miRNAs; U6 snRNA was used as the endogenous reference gene. (**b**) Relative expression levels of lncRNAs; 18S rRNA was used as the endogenous reference gene. (**c**) Relative expression levels of mRNAs. (**d**) Relative expression levels of circRNAs; 18S rRNA was used as the endogenous reference gene. The standard error of the mean for three replicates is represented by the error bars. Samples marked with various letters show a significant difference at *p* < 0.05.

We randomly selected 10 miRNAs, 15 lncRNA, 20 mRNAs, and 7 circRNAs to confirm the reliability of the sequencing technology. We found that 10 miRNAs, 14 lncRNAs, 18 mRNAs, and 7 circRNAs were consistent with the trend of seq results (Figure 4). The result indicated that our seq results were reliable. PCR was performed to confirm our identification circRNAs, the validation back-spliced junction sites were through the sequencing of PCR products. Our result indicated that the back-spliced junction sites have been validated (Figure S5).

## 3. Discussion

In the past decades, transcriptomes and miRNAs have been investigated to help understand the mechanisms of phytoplasma pathogenicity in phytoplasma-infected plants, especially in Paulownia [6,7,10,11,12,27]. Recently, lncRNAs have been investigated in many plants under biotic stresses [19]. In this study, we identified 229 DELs that may be PaWB-related lncRNAs. CircRNAs, a novel type of non-coding RNAs, are ubiquitously expressed in eukaryotic cells during post-transcriptional processes and may play roles in antiviral immunity. In plants, circRNAs have been found to be involved in the response to biotic stresses [39]. In this study, 1634 circRNAs and 65 DECs were found.

The biological functions of lncRNAs and circRNAs have become a hotspot of scientific research in recent years. A growing body of evidence shows that they act as miRNA sponges and fulfill a regulatory function in gene expression [40]. In this study, we identified mRNAs, lncRNAs, circRNAs, and miRNAs in the PF and PFI libraries, and attempted to identify PaWB-related mRNAs, lncRNAs, circRNAs, and miRNAs. To do this, we constructed a ceRNA network to try to detect key RNAs that were involved in the response of Paulownia to PaWB.

### 3.1. Part of the ceRNA Network Centered on pf-miR156g and pf-miR172i

The part of the network centered on pf-miR156g and pf-miR172i contained most of the DEGs that we used to construct the network, including gibberellin 20-oxidase (*Ga20-O*, EVM0006531.1), auxin influx carrier (*AUX1*, EVM0020358.1), ubiquitin-conjugating enzyme E2 D (UBE2D, EVM0023919.1), branchedchain amino acid aminotransferase (*ilvE*, EVM0028155.1), G-type lectin *S*-receptor-like serine/threonine-protein kinase (GsSRK, EVM0021725.1), chlorophyllide a oxygenase (*CAO*, EVM0016969.1), elongation factor G (*EF-G*, EVM0026446.1), calreticulin (*CALR*, EVM0006496.1), CASP-like protein 2B1(*CASPL*, EVM0020959.1), and protein TRIGALACTOSYLDIACYLGLYCEROL 2 (TGD2, EVM0016582.1). The expression of these genes is shown in Figure S6. Gibberellin (GA) [41] and auxin [42] have long been known to play pivotal roles in plant cell expansion or elongation. Ga20-O is involved in GA biosynthesis [43]. GA induced protein have been found in a previous PaWB related study [5], and GA might be related to witches’ broom disease of Paulownia [18]. AUX1 is involved in auxin signal transduction, and, in Arabidopsis, AUX1 is involved in the hook exaggeration phenotype [44]. The two genes encoding Ga20-O and AUX1, which are both involved in phytohormone biosynthesis and signal transduction, may be related to the PaWB-phenotype (bud swelling and witches’ broom). UBE2D is involved in protein processing in the endoplasmic reticulum, and ilvE is involved in amino acid metabolism. EF-G is a guanosine triphosphatase that plays a crucial role in the translocation of tRNAs and mRNAs during ribosomal translation [45]. CALR is an endoplasmic reticulum luminal Ca^2+^-buffering chaperone and is also involved in the folding of newly synthesized proteins and glycoproteins [46]. The differential expression of the four genes encoding these proteins, suggests that protein processing and amino acid metabolism may have been disturbed in the phytoplasma-infected Paulownia. Similar results have been found in other phytoplasma-infected plants [47,48]. During the formation of thylakoids, TGD2 plays an important role and belongs to the phosphatidic acid/lipid transport complex and taken apart in the lipid transfer [49].

### 3.2. Part of the ceRNA Network Centered on pf-miR8767a and pf-miR8767b

This part of the network contained Magnesium chelatase subunit D (ch1D EVM0007798.1), which is involved in porphyrin and chlorophyll metabolism. CAO, which is also in the part centered on pf-miR156j and pf-miR172h, is also involved in porphyrin and chlorophyll metabolism. This result indicates that these two parts of the network may be related to porphyrin and chlorophyll metabolism. Acid phosphatase (PHO, EVM0012688.1) is involved in riboflavin metabolism. Riboflavin is an essential precursor of flavin adenine dinucleotide and flavin mononucleotide co-enzymes. Flavins are co-factors that are integral parts of the redox active sites of enzymes involved in dehydrogenation reactions, dioxygen activation, and electron transfer reactions. Major latex proteins (MLPs) are distantly related to a group of pathogenesis-related proteins [50]. MLP-like protein 28(MLP-28, EVM0016967.1) is in this part of the network. In a previous study, MLP-423 and MLP-34 were identified as a PaWB-related protein [5,15]. In this study, up-regulated MLP-28 may have been involved in the response of Paulownia to PaWB phytoplasma.

### 3.3. Part of the ceRNA Network Is Centered on pf-miR172h

Indole-3-acetic acid (IAA) plays an important role in the regulation of homeostasis, polar transport, and auxin responses. IAA-methyltransferase-1 (IAMT1) could convert IAA to methyl-IAA and IAMT1 is an essential regulator of leaf development [51]. IAMT1 (EVM0021401.1) was found in this part of the network. IAMT1 may play a critical role in IAA homeostasis across a wide range of plants [52]. Hormonal imbalances triggered by phytoplasma infection have been found in previous studies and are considered to be involved in the formation of symptoms such as witches’ broom and short internodes [53]. Metallochaperones are key proteins for the safe transport of metallic ions inside the cell. Genes encoding heavy metal associated isoprenylated plant proteins (HIPPs, EVM0015501.1), which are metallochaperones, were found in this part of the network. HIPP may be involved in various roles in plant development and defense responses [54].

### 3.4. A Hypothetical Model for PaWB

**Figure 5:**
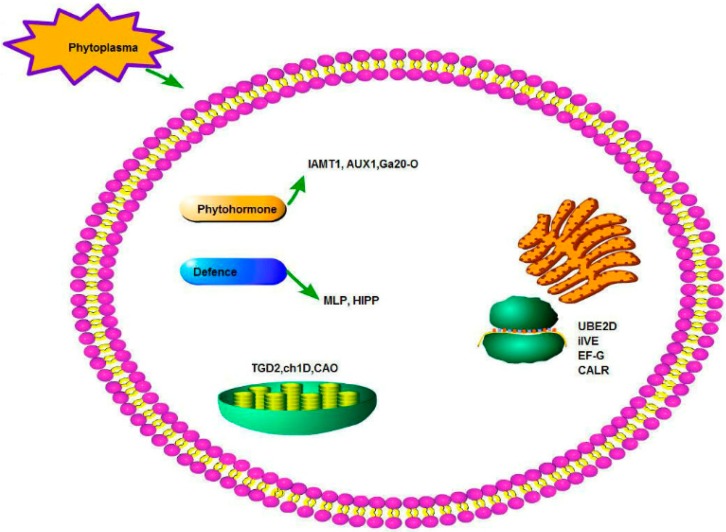
A hypothetical model for PaWB.

Based on our ceRNA network, a hypothetical model for PaWB is proposed (Figure 5). Namely, the phytoplasma influences the plant’s phytohormones (IAA, GA and Auxin), chloroplasts, proteins and amino acids, and defense responses. GA [17] and auxin [8,29,48] were influenced in infected Paulownia. Auxin was suggested to influence symptom expression and phytoplasma colonization in periwinkle infected with periwinkle leaf yellowing phytoplasma [55]. The genes and proteins which were related to GA have been found in phytoplasma-infected Paulownia [5,17]. The influence of chloroplasts may be related to chlorosis of leaves and changes of photosynthesis, as has been found in infected Paulownia [15]. Protein processing and amino acid metabolism were also changed in phytoplasma-infected Paulownia [15]. This may produce amino acids that can help phytoplasmas colonize the host plants. The two defense genes (*MLP-28* and *HIPP*) may be involved in plant–pathogen interactions with an unknown pattern.

## 4. Materials and Methods

### 4.1. Plant Materials

All the biological materials used in this study were obtained from the Institute of Paulownia, Henan Agricultural University, China. Two groups of *P. fortunei* seedlings were set up: healthy (PF) and PaWB-infected (PFI). The cultivation procedures were as described by Fan et al. [9]. The terminal buds from three individual plants were combined to form one biological replicate, and three biological replicates were used for each group.

### 4.2. Phytoplasmas Detection

PaWB phytoplasma was detected by nested-PCR according to the method of Lee et al. [56]. The PCR procedure and agarose gel electrophoresis were performed as described by Fan et al. [57].

### 4.3. RNA Library Construction and Sequencing

Total RNA was extracted using a Trizol reagent (Invitrogen, Carlsbad, CA, USA). The total RNA quantity and purity were analyzed using a Bioanalyzer 2100 (Agilent, Santa Clara, CA, USA). Approximately 1 µg of total RNA was used to prepare the small RNA (sRNA) PF and PFI libraries according to the protocol of a TruSeq Small RNA Sample Prep Kit (Illumina, San Diego, CA, USA). We constructed six sRNA libraries (PF-1,2,3 and PFI-1,2,3) from the three biological replicates for each group. We performed single-end sequencing on an Illumina HiSeq 2500 platform at the LC-BIO following the manufacturer’s recommended protocol.

For the lncRNAs, mRNAs, and circRNAs, approximately 10 µg of the total RNA representing a specific adipose type was used to deplete ribosomal RNA according to the Epicentre Ribo-Zero Gold Kit (Illumina) instructions. Following purification, the poly(A)− or poly(A)+ RNA fractions were fragmented using divalent cations under elevated temperature. The cleaved RNA fragments were reverse transcribed to create the final cDNA libraries in accordance with the protocol for the mRNA-Seq Sample Preparation Kit. The average insert size for the paired-end libraries was 300 bp. We performed the paired-end sequencing on an Illumina HiSeq 4000 platform at the LC-BIO (Hangzhou, China) following the manufacturer’s recommended protocol.

### 4.4. Small RNA Sequencing, miRNA Identification, and the Prediction of miRNA Target Genes

The raw reads were filtered using an in-house program (ACGT101-miR) to remove adapter dimers, junk, low complexity sequences, common RNA families (rRNA, tRNA, snRNA, snoRNA), and repeats. Subsequently, unique sequences 18–25 nucleotide in length were searched against miRBase 21.0 to identify known miRNAs and novel miRNAs. Length variations at both the 3′ and 5′ ends and one mismatch within the alignment were allowed. The mapped sequences were identified as known miRNAs. These mapped pre-miRNAs were used in BLAST searches against the Paulownia genome to determine their genomic locations. To identify the novel predicted miRNAs, the remaining unmapped sequences were aligned to the Paulownia genome by BLAST, and the hairpin RNA structures of the mapped sequences with 120 nt flanking sequences were predicted using RNAfold software (http://rna.tbi.univie.ac.at/cgibin/RNAfold.cgi). The criteria for secondary structure prediction were similar to a previous study [58].

To predict the genes targeted by the most abundant miRNAs, target prediction algorithms (Target Finder 50) were used to identify miRNA binding sites. The most abundant miRNAs targets were annotated using the gene ontology (GO) and the Kyoto Encyclopedia of Genes and Genomes (KEGG) Pathway databases.

### 4.5. Genome Mapping and Transcript Assembly

Cutadapt [59] was used to remove reads that contained adaptor sequences, low quality bases, or undetermined bases. Sequence quality was verified using FastQC (http://www.bioinformatics.babraham.ac.uk/projects/fastqc/). We used Bowtie2 [60] and TopHat2 [61] to map the clean reads to the Paulownia genome. The mapped reads in the two libraries were assembled using StringTie [62]. Then, the transcriptomes of the PF and PFI samples were merged using Perl scripts to reconstruct a comprehensive transcriptome. StringTie [62] and Ballgown [63] were used to estimate the expression levels of the transcripts in the final transcriptome dataset.

### 4.6. lncRNA Identification

Transcripts that overlapped with known mRNAs and transcripts < 200 bp in length were discarded. Then, the Coding Potential Calculator (CPC [64]) and Coding–Non-Coding Index (CNCI [65]) were used to predict transcripts with coding potential. All transcripts with CPC score < −1 and CNCI score < 0 were removed. The remaining transcripts with class codes (i, j, o, u, or x) were considered as lncRNAs.

### 4.7. Differential Expression Analysis of mRNAs and lncRNAs

StringTie [62] was used to calculate the expression levels of the mRNAs and lncRNAs using FPKM. Differentially expressed mRNAs and lncRNAs were defined as having log2 (fold change) > 1 or < −1 and statistical significance (*p* value < 0.05) using the Ballgown R package.

### 4.8. Target Gene Prediction and Functional Analysis of lncRNAs

To explore the functions of the lncRNAs, we predicted the *cis*-target genes (neighboring genes) of the identified lncRNAs. We selected coding genes that were 100,000 bp upstream and downstream of the identified lncRNAs using a Python script. RNAplex was used to calculated the energy of potential mRNA–lncRNA interactions [19]. We performed a functional analysis of the candidate lncRNA target genes using Blast2GO. Significance was defined as *p* value < 0.05.

### 4.9. CircRNA Identification

Cutadapt [59] was used to remove the reads that contained adaptor sequences, low quality bases, and undetermined bases. Sequence quality was verified using FastQC. We used Bowtie2 [60] and TopHat2 [61] to map the reads to the Paulownia genome. Remaining reads were mapped to the genome using TopHat-Fusion [66]. CIRCExplorer [67,68] was used to de novo assemble the mapped reads to predict circular RNAs. Then, back-splicing reads were identified among the unmapped reads by TopHat-Fusion [66] and CIRCExplorer [68]. Unique circRNAs were detected in both libraries and their expressions were calculated using in-house scripts. Differentially expressed circRNAs between the two libraries were defined as having *p* value < 0.05 using the R package edgeR [69].

### 4.10. Validation of ceRNAs

To confirm the seq results, qRT-PCR detection was performed to evaluate the expression levels of, miRNAs, lncRNAs, and mRNAs using a SYBR Green PCR kit (GeneCopoeia, Inc., Rockville, MD, USA) with ViiA™ 7 Dx platform (ABI, Foster City, CA, USA). The primers for lncRNAs and mRNAs were designed using Primer Express 3.0 (Applied Biosystems, Stockholm, Sweden) and the specific stem-loop primers for the miRNAs were designed as reported previously [70]. For miRNAs, the U6 snRNA gene was chosen as the endogenous control; for lncRNAs and mRNAs, Paulownia 18S rRNA was used as the endogenous reference gene. CircRNA were validated by PCR and sanger sequencing according the methods in previous studies [71,72]. All qRT-PCR amplifications were carried out in triplicate, with the standard reaction program. The generated real-time data were analyzed according a previous study [73]. The primers used are listed in Table S1.

## 5. Conclusions

In this study, RNA sequencing was performed for heathy and infected *Paulownia fortunei*. Among a total of 3126 lncRNAs, 1634 circRNAs, and 550 miRNAs that were identified, 229 lncRNAs, 65 circRNAs, and 65 miRNAs were differentially expressed in a significant manner. We constructed the first ceRNA network in Paulownia comprising 5 miRNAs, 4 circRNAs, 5 lncRNAs, and 15 mRNAs, all of which were differentially expressed. The genes in the ceRNA network were involved mainly in phytohormone biosynthesis and signal transduction, protein processing and amino acid metabolism, chloroplast, and defense responses.

## Supplementary Materials

Supplementary materials can be found at http://www.mdpi.com/1422-0067/19/8/2463/s1.

## Abbreviations

lncRNA	Long noncoding RNA
circRNA	Circular RNA
miRNA	MicroRNA
PaWB	Paulownia witches’ broom
ceRNA	Competing endogenous RNA
GO	Gene ontology
KEGG	Kyoto Encyclopedia of Genes and Genomes
CPC	Coding Potential Calculator
CNCI	Coding–Non-Coding Index
DECs	Differentially expressed circRNAs
DELs	Differentially expressed lncRNAs
DEGs	Differentially expressed genes
DEMs	Differentially expressed miRNA
AIP	Auxin-induced protein PCNT115-like
OPR	12-oxophytodienoic acid reductase
PP2C	Probable protein phosphatase 2C 40
CAD	Cinnamyl-alcohol dehydrogenase
ACSL	Long-chain acyl-CoA synthetase
Ses	Secologanin synthase-like
gpx	Glutathione peroxidase
CAO	Chlorophyllide a oxygenase
Ga20-O	Gibberellin 20-oxidase
AUX1	Auxin influx carrier
UBE2D	Ubiquitin-conjugating enzyme E2 D
ilvE	Branchedchain amino acid aminotransferase
GsSRK	G-type lectin S-receptor-like serine/threonine-protein kinase
EF-G	Elongation factor G
CALR	Calreticulin
CASPL	CASP-like protein 2B1
TGD2	Protein TRIGALACTOSYLDIACYLGLYCEROL 2
GA	Gibberellin
ch1D	Magnesium chelatase subunit D
PHO	Acid phosphatase
MLPs	Major latex proteins
IAA	Indole-3-acetic acid
IAMT1	IAA-methyltransferase-1
HIPPs	Heavy metal-associated isoprenylated plant proteins
