# Supplementary material for: ceRNA Cross-Talk in Paulownia Witches’ Broom Disease
Source: Int J Mol Sci. 2018 Aug 20;19(8):2463. doi: 10.3390/ijms19082463 (PMC6121691; doi:10.3390/ijms19082463)
Supplement: Supplementary file 1 [file ijms-19-02463-s001.pdf]

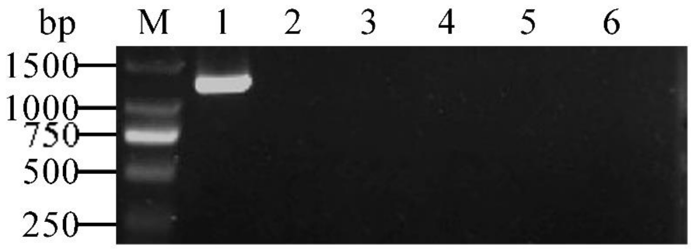

Figure S1 Detection of phytoplasma in Paulownia seedings. (1) PFI; (2) PF; (3) ddH2O; (M) DNA Marker.

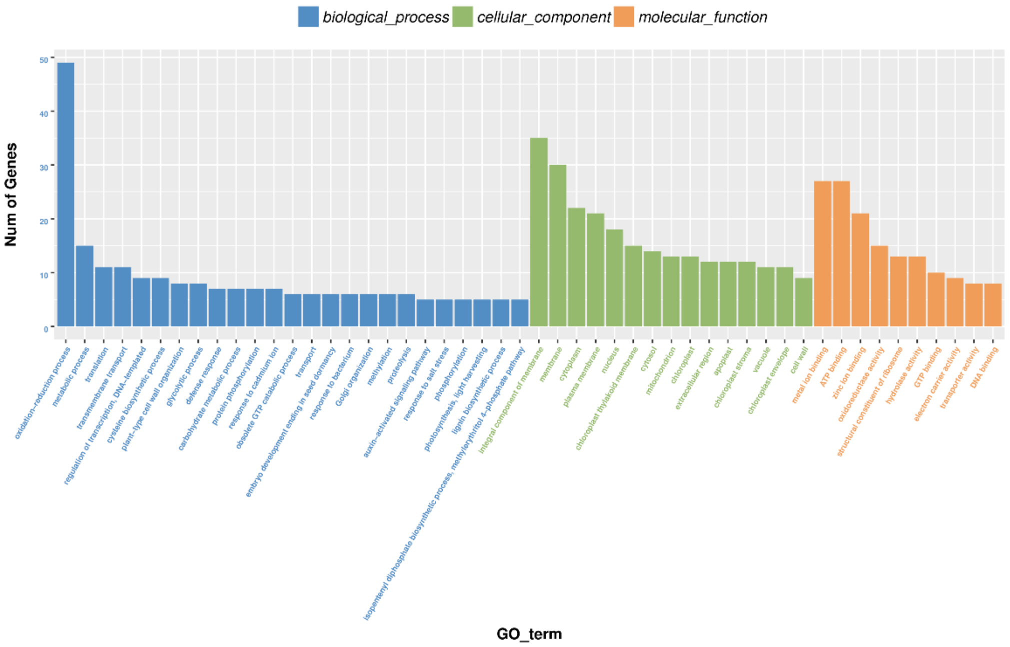

Figure S2 GO analysis of differentially expressed genes.

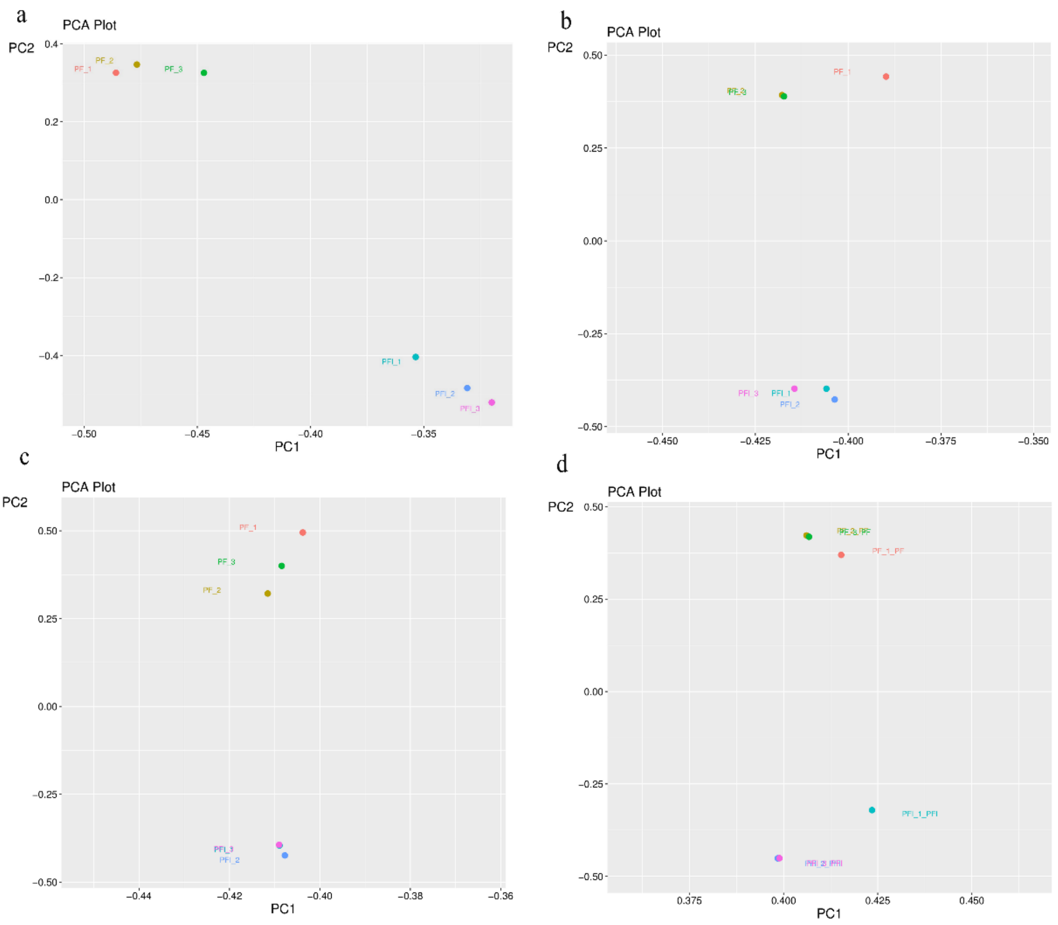

Figure S3 The principal component analysis (PCA) of differentially regulated miRNA, mRNA, lncRNAs, and circRNAs. (a) PCA for miRNA, (b) PCA for mRNA, (c) PCA for lncRNAs, (d) PCA for circRNAs.

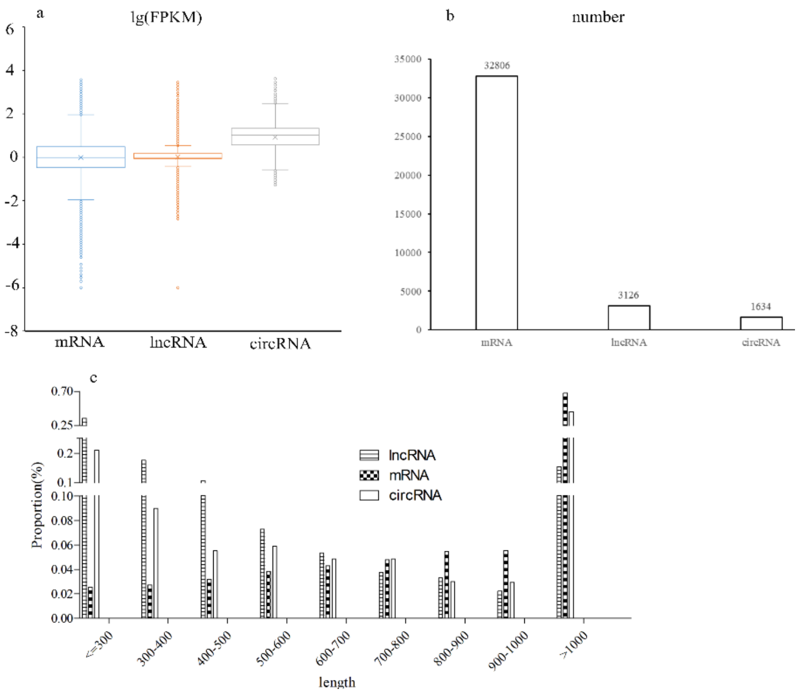

Figure S4 Comparison between mRNA, lncRNA, and circRNA. (a) comparison of expression level, (b) comparison of identified number, (c) comparison of length distribution.

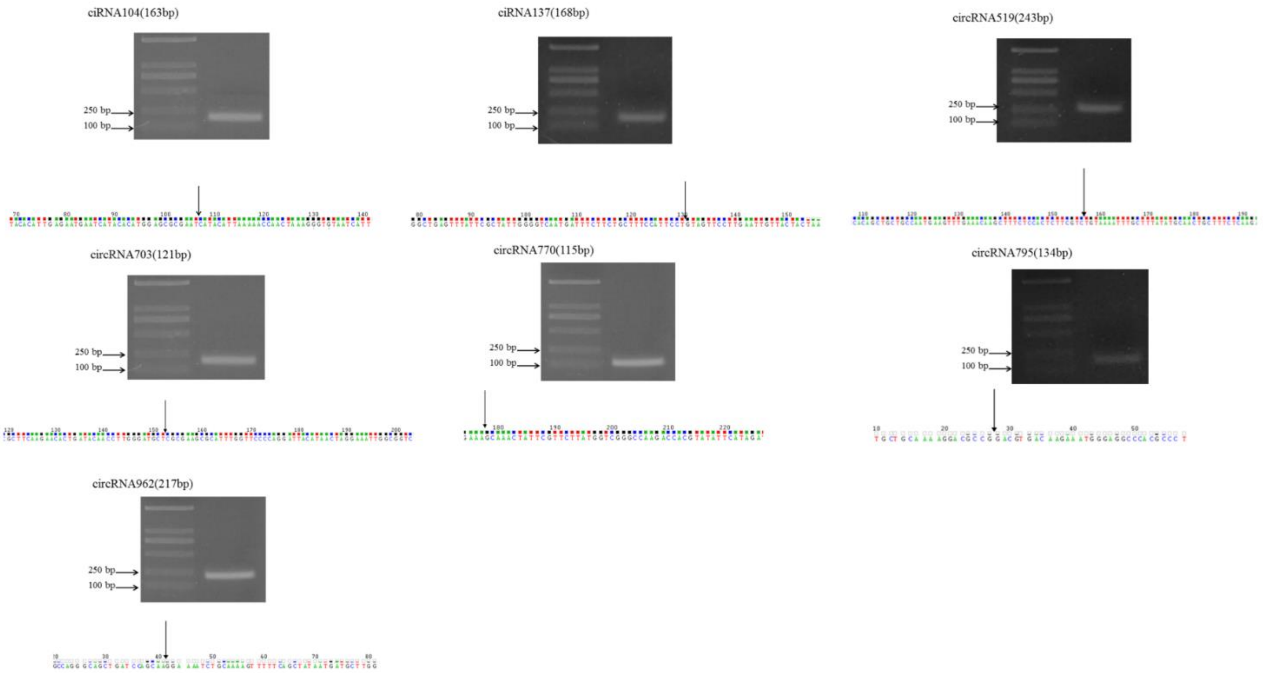

Figure S5 Experimental validation of circRNAs in Paulownia.

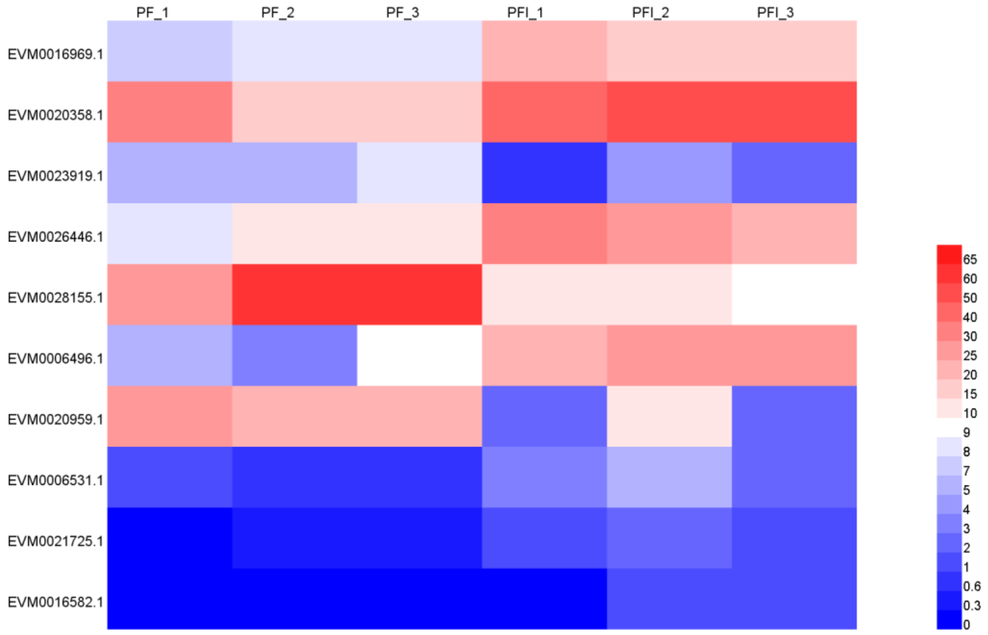

Figure S6 Heatmap of genes in the ceRNA network which are centered on pf-miR156g and pf-miR172i.
